# *TP63*‐mutation as a cause of prenatal lethal multicystic dysplastic kidneys

**DOI:** 10.1002/mgg3.1486

**Published:** 2020-09-02

**Authors:** Isabel Friedmann, Carla Campagnolo, Nancy Chan, Ghislain Hardy, Maha Saleh

**Affiliations:** ^1^ Schulich School of Medicine and Dentistry University of Western Ontario London ON Canada; ^2^ Division of Genetics and Metabolism Department of Paediatrics London Health Sciences Centre London ON Canada; ^3^ Department of Pathology London Health Sciences Centre London ON Canada; ^4^ Department of Obstetrics and Gynecology London Health Sciences Centre London ON Canada

**Keywords:** antenatal ultrasound, ectrodactyly‐ectodermal dysplasia‐clefting syndrome 3, renal dysplasia, tumor protein p63, whole‐exome sequencing

## Abstract

**Background:**

Ectrodactyly‐ectodermal dysplasia‐clefting syndrome 3 (EEC) is one of the six overlapping syndromes caused by mutations in the tumor protein p63 gene (*TP63*). EEC is suspected when patients have cleft hands or feet, polydactyly, and syndactyly, abnormal development of the ectodermally derived structures, and orofacial clefting. Genitourinary (GU) anomalies have been identified in patients with EEC, yet these are often under‐recognized and under‐reported. The available literature on sonographic prenatal findings is sparse, especially when considering GU anomalies.

**Methods:**

We present the case of a male stillborn fetus, who was found antenatally to have multicystic dysplastic kidneys and anhydramnios. Following the termination of pregnancy, examination and autopsy further revealed unilateral polydactyly and bilateral syndactyly which had not been previously identified on antenatal ultrasound.

**Results:**

Whole‐exome sequencing (WES) revealed a de novo heterozygous pathogenic variant in exon 5 of the *TP63* gene: p.His247Arg: c.740A>G (NM_003722.4) which has been reported in the literature. The His247Arg variant has been published as a pathogenic variant in association with EEC, both with and without orofacial clefting.

**Conclusion:**

Our prenatal case expands the phenotypic spectrum of *TP63*‐related disorders in general. In addition, it adds to the phenotype associated with the His247Arg pathogenic variant responsible for EEC. Further, we highlight the importance of WES as a postnatal tool to help clarify unexpected findings, and as a way to add to the spectrum of existing phenotypes of known single‐gene disorders.

## Graphical abstract

In this paper, we describe a prenatal presentation of multicystic dysplastic kidneys and anhydramnios in a male fetus with a postnatal autopsy finding of unilateral upper limb polydactyly and bilateral syndactyly. Whole exome sequencing (WES) revealed a de novo heterozygous pathogenic variant in the TP63 gene: p.His247Arg: c.740A>G (NM_003722.4), which has been reported with ectrodactyly‐ectodermal dysplasia‐clefting syndrome 3 (EEC), both with and without orofacial clefting.

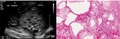

## 1 INTRODUCTION

Ectrodactyly‐ectodermal dysplasia‐clefting syndrome 3 (EEC) is one of at least six overlapping syndromes caused by mutations in the tumor protein p63 gene (*TP63*) (Rinne, Hamel, van Bokhoven, & Brunner, 2006). EEC is often suspected when patients have any combination of cleft hands or feet, polydactyly, and syndactyly, abnormal development of the ectodermally derived structures including hair, teeth, skin, and nails, and cleft lip and/or palate (Rinne, Brunner, & van Bokhoven, 2007). Genitourinary (GU) anomalies are often found in patients with EEC, yet these are often under‐recognized and under‐reported (Hyder, Beale, O'Connor, & Clayton‐Smith, 2017). Further, the available literature on antenatal ultrasound findings in patients with EEC is sparse to begin with, and only a handful of articles discuss antenatal findings of those with GU anomalies. Herein, we present the case of a male stillborn fetus, who was noted antenatally to have multicystic dysplastic kidneys and anhydramnios. Given the poor outcome and high likelihood of lethality, a therapeutic termination of pregnancy was offered, with consent to full autopsy. Postnatal examination and autopsy results further revealed unilateral hand polydactyly and bilateral hand syndactyly, which had not been previously identified on antenatal ultrasound. The findings were suggestive of a split hand deformity. In addition, there was no evidence of cleft lip and palate deformity. Whole‐exome sequencing (WES) revealed a de novo heterozygous pathogenic variant in exon 5 of the *TP63* gene: p.His247Arg: c.740A>G (NM_003722.4) which has been reported as a pathogenic variant in association with EEC, both with and without orofacial clefting (Clements, Techanukul, Coman, Mellerio, & McGrath, 2010).

Our prenatal case expands the phenotypic spectrum of *TP63*‐related disorders. In addition, it adds to the phenotype associated with the His247Arg pathogenic variant responsible for EEC. Further, we highlight the importance of WES as a postnatal tool to help clarify unexpected findings, and as a way to add to the spectrum of existing phenotypes of known single‐gene disorders.

### 1.1 Case presentation

**FIGURE 1:**
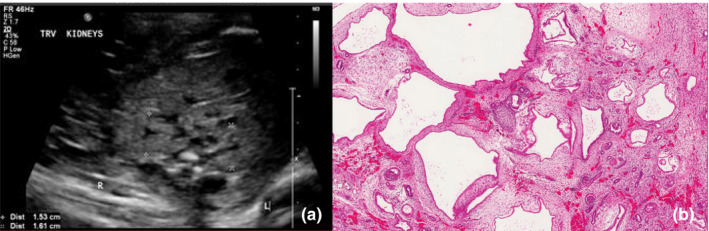
(a) Antenatal ultrasound image depicting a transverse view of the fetus’ bilateral multicystic dysplastic kidneys. (b) Photomicrograph demonstrating multicystic renal dysplasia; variably sized cysts, collarettes of mesenchyme, immature glomeruli, and central cartilage (hematoxylin and eosin staining).

At the time of our first encounter, our patient was a 25‐year‐old primigravida female at 22 + 4 weeks of gestational age. Pregnancy had been largely unremarkable aside from concerning findings noted on antenatal anatomy scans. The ultrasounds had revealed a fetus with bilateral enlarged and echogenic kidneys, multiple cysts within the renal parenchyma, anhydramnios, and a non‐visualized bladder (Figure 1). Integrated prenatal screening had been performed, and reported as a negative screen for Trisomy 21 and Trisomy 18. A three‐generation family history was obtained. Our patient was of German and Scottish descent, and her partner was of Irish and English descent, with no known consanguinity. The family history was negative for any other known congenital anomalies and was otherwise unremarkable.

**FIGURE 2:**
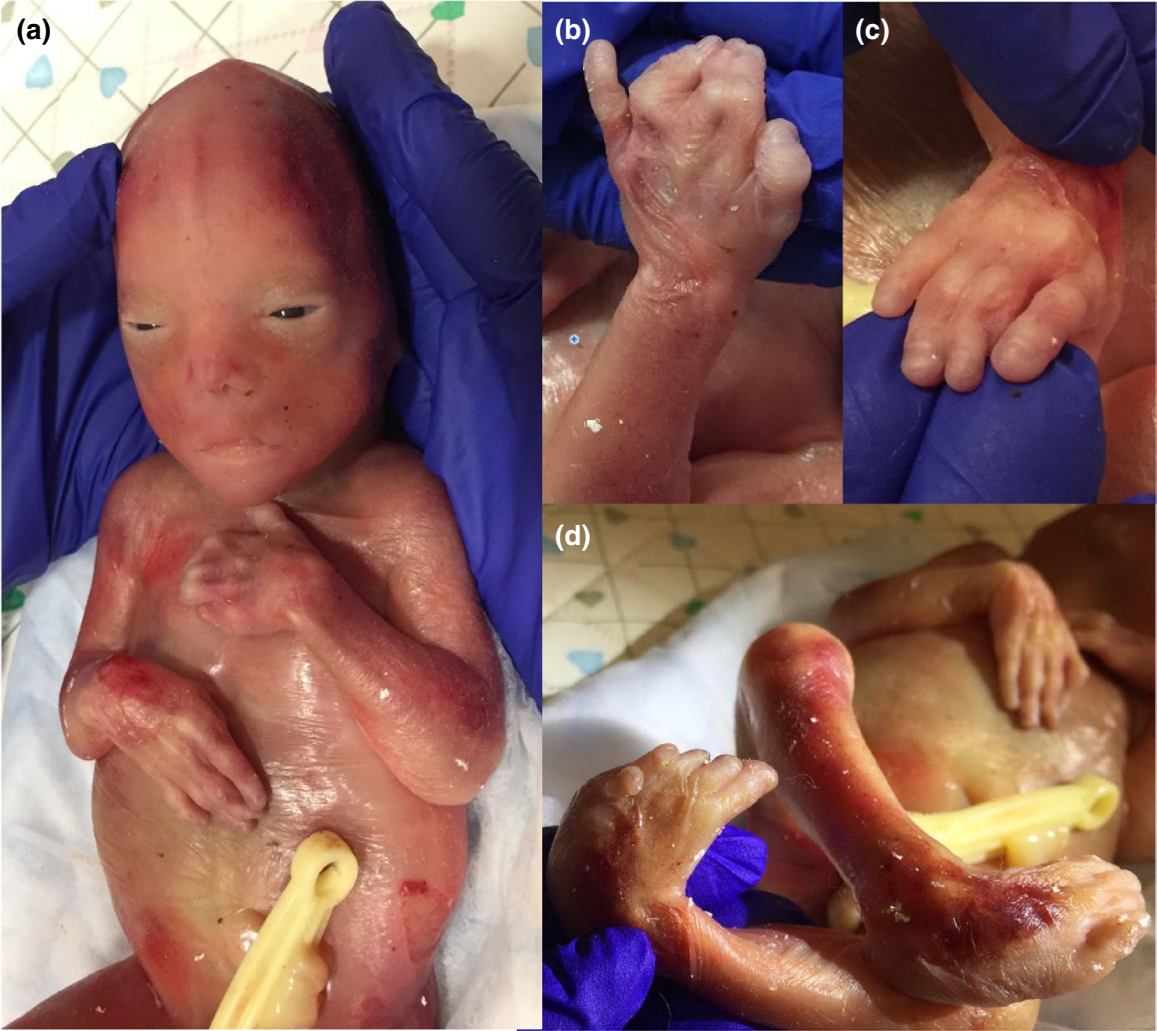
(a) Facies consistent with Potter sequence, and distal extremity anomalies noted on external examination. (b) Post‐axial polydactyly of the left hand (six digits) with syndactyly between digits two and three, and four and five. (c) Partial syndactyly of the right hand between digits three and four. (d) Bilateral clubfeet (versus positional changes).

Due to overall poor prognosis for the fetus, and likely incompatibility with life, therapeutic termination of pregnancy was initiated with the induction of labor. Following the delivery of a male stillborn fetus at 22 + 5 weeks of gestational age, external examination and autopsy were performed. Upon examination, the male fetus had evidence of facies consistent with Potter sequence. A high‐arched palate was identified, as was maxillary hypoplasia, and there was no evidence of any orofacial clefting. There were severe elbow contractures, and elbow pterygia were present bilaterally. There was post‐axial polydactyly of the left hand (six digits), as well as full cutaneous syndactyly between digits two and three, and four and five. There was partial syndactyly of the right hand between digits three and four. There was no polydactyly or syndactyly of the toes. Bilateral clubfeet could not be excluded, with positional changes being considered as well. Skin examination was in keeping with the gestational age of the fetus and was otherwise unremarkable. Aside from the maxillary hypoplasia and aforementioned syndactyly, no other classic features of the EEC spectrum were identified (Figure 2).

Autopsy confirmed the presence of pulmonary hypoplasia, in the presence of bilaterally enlarged kidneys with multicystic renal cortical dysplasia. The right kidney weighed 8 g, and the left weighed 5 g, with a combined weight of 13 g (the combined age‐related norm was listed as 4.77 ± 1.39 g on autopsy report). The cysts were disorganized, variable in shape and size, and were as large as 0.5 cm in diameter. They were lined by simple cuboidal epithelium and many had collars of smooth muscle. The ureters and bladder were clearly identified with no obvious abnormalities (Figure 1). The testes were both intra‐abdominal and consistent with the gestational age of the stillborn fetus.

Skeletal survey was performed and was negative for any obvious skeletal congenital anomalies beyond the post‐axial polydactyly of the left hand.

Differential diagnosis at the time, based on the above findings, was quite broad. Considerations included triploidy, microdeletion and microduplication syndromes, and single‐gene disorders including ciliopathies, and other congenital anomalies of the kidneys and urinary tract (CAKUT). QF‐PCR testing was performed in the stillborn fetus and was consistent with a normal disomic complement of chromosomes 13, 18, and 21 in a male fetus. Chromosomal microarray results were also consistent with a normal male fetus.

A whole‐exome trio (XomeDx) was submitted clinically. The exonic regions and flanking splice junctions of the genome were sequenced by massively parallel (NextGen) sequencing on an Illumina sequencing system with a minimum of 100 bp paired‐end reads. The reads were aligned to the human genome build GRCh37/UCSC hg19 and analyzed for sequence variants using Xome Analyzer. This identified a de novo heterozygous pathogenic variant in exon 5 of the *TP63* gene: p.His247Arg: c.740A>G (NM_003722.4).

This particular missense variant has been reported in several individuals affected with EEC (Clements et al., 2010; Hyder et al., 2017; Polla et al., 2015; Rinne et al., 2006), including two siblings affected by severe ectrodactyly and mild ectodermal dysplasia, without clefting (Sorasio, Biamino, Garelli, Ferrero, & Silengo, 2009). Associated GU anomalies have also been described, including bilateral hydronephrosis (Hyder et al., 2017), congenital absence of the right kidney with left hydronephrosis in one individual, and mild vesicoureteric reflux in another (Clements et al., 2010).

## 2 DISCUSSION

EEC is an autosomal dominant condition that is one of at least six overlapping syndromes caused by mutations in the *TP63* gene located on chromosome 3q27 (Rinne et al., 2006). Ectrodactyly is often manifested as split hands and feet, due to the lack of one or more central digits, as well as syndactyly. Ectodermal dysplasia refers to abnormal growth or development of any of the structures derived from the embryonal ectoderm, namely skin, hair, teeth, nails, and sweat and sebaceous glands. Finally, clefting refers predominantly to the orofacial clefting seen in EEC, which manifests as cleft lip and/or palate (Rinne et al., 2007). Hyder et al., (2017) describe that there is significant variable expressivity in EEC, to the extent that the diagnosis can be considered even if the three classic elements are not all present. As well, there are other commonly associated findings, such as blonde and sparse hair, and, in particular, GU anomalies, which often go under‐recognized.

Many authors have reported a wide range of GU anomalies associated with EEC, including but not limited to renal agenesis, hydronephrosis, dilated ureters, bladder diverticula, interstitial nephritis, urethral stenosis, hypospadias, and micropenis (Buss, Hughes, & Clarke, 1995; Hyder et al., 2017; Maas, de Jong, Buss, & Hennekam, 1996; Maclean et al., 2007; Nardi et al., 1992; Rollnick & Hoo, 1988). Further, Clements et al., (2010) report on an adult male patient with the same missense variant as our patient, who was found to have congenital absence of the right kidney and left hydronephrosis.

The available literature on antenatal sonographic findings of patients with EEC is sparse to begin with. Further, the majority of published case reports describe fetuses with classic findings of cleft lip and palate, or clefting of the hands and feet, but no identifiable GU anomalies. (Anneren, Andersson, Lindgren, & Kjartansson, 1991; Bronshtein & Gershoni‐Baruch, 1993; Kohler, Sousa, & Jorge, 1989; Leung, MacLachlan, & Sepulveda, 1995; Rios et al., 2012; Yang, Huang, Han, & Li, 2017).

To our knowledge, only five published articles describe fetuses who went on to receive a diagnosis of EEC, who had GU anomalies detected antenatally. Chuangsuwanich, Sunsaneevithayakul, Muangsomboon, and Limwongse, (2005) describe the case of a male fetus at 24 weeks of gestation who was noted on antenatal ultrasound to have a right nephrogenic cyst measuring approximately 6.0 cm in diameter, in conjunction with a left dysplastic cystic kidney, significant oligohydramnios, and hydrops fetalis. Autopsy following termination of pregnancy also revealed significant pulmonary and bladder hypoplasia, in addition to cleft lip and palate, ectrodactyly and syndactyly of the hands and feet, Potter's facies, and clubfeet. The diagnosis of EEC in this patient was purely clinical. The only genetic testing performed in this stillborn fetus was a karyotype, which was resulted as a normal male. Allen and Maestri, (2008) describe the case of a male fetus with a known family history of EEC, at 17 weeks of gestation, who was noted on antenatal ultrasound to have a left‐sided facial cleft, as well as bilateral ectrodactyly of the hands and feet. Repeat ultrasound at 22 weeks of gestation revealed a highly echogenic left kidney, with plans to better define the renal anomaly postnatally. There is no mention of molecular testing being carried out in this patient. Janssens, Defoort, Vandenbroecke, Scheffer, and Mortier, (2008) report on the case of a fetus with EEC at 16 weeks of gestation, in whom prune belly anomaly was detected antenatally. The clinical diagnosis of EEC was also made in the fetus's mother, who had bilateral cleft lip and palate, syndactyly, ureteropelvic junction stenosis, and hymenal stenosis. Molecular diagnosis of EEC was ultimately confirmed in both patients, with the identification of a heterozygous mutation in *TP63*: p.Arg243Trp: c.727C>T (NM_003722.5). Enriquez, Krivanek, Flottmann, Peters, and Wilson, (2016) describe two separate sibling fetuses who had classic findings of EEC in addition to GU anomalies noted on antenatal ultrasound. More specifically, the first sibling was noted at 18 weeks of gestation to have megaureters with significant bilateral hydronephrosis, marked oligohydramnios, and an undetectable bladder. A formal autopsy was not performed on this sibling following termination of pregnancy at 20 weeks of gestation. The second sibling was noted at 12 weeks of gestation to have abnormal bladder distension, suggesting urethral obstruction. Repeat ultrasound at 14 weeks of gestation revealed megacystis and significant abdominal distension. Following the termination of pregnancy, autopsy further revealed urethral outflow obstruction as a product of a patent proximal urethra ending in a blind pouch, in addition to a rectovaginal fistula, ambiguous genitalia, and an imperforate anus. Chromosomal microarray was performed on the first sibling and was resulted as a normal male. There was not enough DNA available from the second sibling to perform a microarray; however, microsatellite analysis detected the presence of both an X and a Y chromosome. Molecular diagnosis of EEC was confirmed in both patients, with the identification of a heterozygous mutation in *TP63*: p.Asp351Asn: c.1051G>A (NM_003722.4), which was not detected in either parent.

Finally, Hyder et al., (2017) describe the case of a male fetus noted to have bilateral cleft lip and palate during his 20‐week antenatal ultrasound, and later found to have bilateral hydronephrosis on repeat ultrasound ten weeks later. On follow‐up postnatally, the patient was found to have progressive ureteric dilation up to the pelvicalyceal system, without vesicoureteric reflux, and progressive urethral narrowing, ultimately requiring surgical intervention. Diagnosis of EEC was confirmed postnatally and the His247Arg pathogenic variant in *TP63* was identified (Table 1).

**TABLE 1:** Review of the literature describing fetuses identified antenatally to have GU anomalies, who went on to receive a diagnosis of EEC.

Case report	Fetal sex and age at presentation	Antenatally‐detected GU‐anomalies	Postnatal GU anomalies	Non‐GU anomalies	Molecular finding
Our patient	‐Male fetus seen at 22 weeks of gestation	‐Bilaterally enlarged and echogenic kidneys, multiple cysts within the renal parenchyma, anhydramnios, non‐visualized bladder	‐Autopsy revealed bilaterally enlarged kidneys with multicystic renal cortical dysplasia, multiple, largest 0.5 cm in diameter, normal ureters and bladder	‐Pulmonary hypoplasia ‐Potter's facies ‐High‐arched palate, maxillary hypoplasia ‐Left hand poly/syndactyly ‐Right hand syndactyly ‐Clubfeet	‐De novo heterozygous mutation (p.His247Arg) in *TP63*
Hyder et al. (2017)	‐Male fetus seen at 20 and 30 weeks of gestation	‐Bilateral hydronephrosis identified at 30 weeks of gestation	‐Postnatal examination revealed progressive ureteric dilation up to the pelvicalyceal system, without vesicoureteric reflux, and progressive urethral narrowing requiring surgical intervention	‐Bilateral cleft lip and palate	‐Heterozygous mutation (p.His247Arg) in *TP63*
Enriquez et al. (2016)	‐Fetus (sibling) 1 seen at 18 weeks of gestation ‐Fetus (sibling) 2 seen at 12 and 14 weeks of gestation	‐Fetus 1 identified to have megaureters, significant bilateral hydronephrosis, marked oligohydramnios, undetectable bladder ‐Fetus 2 identified to have abnormal bladder distension at 12 weeks of gestation; follow‐up at 14 weeks revealed megacystis and significant abdominal distension	‐Autopsy of Fetus 2 revealed urethral outflow obstruction as a product of a patent proximal urethra ending in a blind pouch ‐Autopsy also revealed rectovaginal fistula, ambiguous genitalia, and imperforate anus	‐Ectrodactyly ‐Bilateral cleft lip and palate for both fetuses	‐Heterozygous mutation (p.Asp351Asn) in *TP63* confirmed in both fetuses
Allen et al. (2008)	‐Male fetus seen at 17 and 22 weeks of gestation	‐Highly echogenic left kidney identified at 22 weeks of gestation	‐Renal dysplasia on postnatal ultrasound	‐Left‐sided facial cleft ‐Bilateral ectrodactyly of hands and feet	‐No molecular confirmation
Janssens et al. (2008)	‐Fetus seen at 16 weeks of gestation	‐Prune belly anomaly detected	‐Mother had ureteropelvic junction stenosis and hymenal stenosis	‐Cleft lip and palate and ectrodactyly for mother and fetus	‐Heterozygous mutation (p.Arg243Trp) in *TP63* confirmed in mother and fetus
Chuangsuwanich et al. (2005)	‐Male fetus seen at 24 weeks of gestation	‐Right nephrogenic cyst, 6.0 cm in diameter, left dysplastic cystic kidney, significant oligohydramnios, and hydrops fetalis	‐Autopsy revealed bladder hypoplasia	‐Pulmonary hypoplasia ‐Potter's facies ‐Cleft lip and palate ‐Ectrodactyly and syndactyly of hands and feet ‐Clubfeet	‐No molecular confirmation

Our patient presented with multicystic dysplastic kidneys and anhydramnios incompatible with life. The postnatal examination was limited to syndactyly and polydactyly of the left hand, and partial syndactyly of the right hand. Given those additional postnatal findings, our family qualified for a clinical WES. Considering the isolated limb findings, negative family history, and lack of orofacial cleft, the diagnosis of EEC would not have been possible without WES. Our family had consented to the termination of pregnancy via induction, and agreed to an autopsy, which helped confirm the diagnosis. In cases of other modes of surgical terminations, pertinent postnatal examination findings may be missed, and a limited gene panel, such as a CAKUT panel, may be offered, thus missing the opportunity to discover such an important molecular diagnosis.

## 3 CONCLUSION

GU anomalies are a common, yet under‐reported and under‐recognized feature of EEC. Our case expands the phenotypic spectrum of *TP63*‐related disorders to include multicystic dysplastic kidneys and anhydramnios as a prenatal lethal presentation of EEC due to the His247Arg pathogenic variant.

Moreover, our experience emphasizes the clinical utility of WES as a postnatal investigation. WES serves as a strong diagnostic tool for prenatal lethal presentations and can help clarify unexpected physical findings on postnatal examination. In addition, WES adds to the spectrum of existing phenotypes of known single‐gene disorders.

## 4 EDITORIAL POLICIES AND ETHICAL CONSIDERATIONS

Written informed consent was obtained from the adult patient described in this manuscript to publish her and her fetus's case.

## CONFLICTS OF INTEREST

None of the authors have any conflicts or potential conflicts of interest to disclose.

## AUTHOR CONTRIBUTIONS

IF and MS drafted the manuscript. CC reviewed the first draft of the manuscript and was in contact with the family to obtain consent. NC and GH reviewed the first draft of the manuscript and contributed to the final content, including images of autopsy and ultrasound. All authors approved the final version of the manuscript.

## Data Availability

The data that support the findings of this study are available from the corresponding author upon reasonable request.
